# An Italian Validation of the 5-Item Attitudes to Mental Illness Questionnaire (AMIQ): A Useful Tool for Rapid Assessment of Stigma, Acceptance, and Tolerance

**DOI:** 10.3390/healthcare12030395

**Published:** 2024-02-03

**Authors:** Lorenzo Stacchini, Marco Fonzo, Alessandro Catalini, Giuseppe Di Martino, Omar Enzo Santangelo, Tiziana Menegon, Fabrizio Cedrone, Vincenza Gianfredi

**Affiliations:** 1 Department of Health Science, University of Florence, 50134 Florence, Italy; lorenzo.stacchini@unifi.it; 2 Hygiene and Public Health Unit, Department of Cardiac-Thoracic-Vascular Sciences and Public Health, University of Padova, 35131 Padova, Italy; marco.fonzo@unipd.it; 3 Food Safety and Nutrition Unit, Local Health Authority of Macerata, 62100 Macerata, Italy; 4 Department of Medicine and Ageing Sciences, “G. d’Annunzio” University of Chieti-Pescara, 66100 Chieti, Italy; giuseppe.dimartino@unich.it; 5 Unit of Hygiene, Epidemiology and Preventive Medicine, ASL Pescara, 65100 Pescara, Italy; 6 Azienda Socio Sanitaria Territoriale di Lodi, ASST di Lodi, 26900 Lodi, Italy; omarenzosantangelo@hotmail.it; 7 Health Promotion Unit, Azienda ULSS Marca Trevigiana, 31100 Treviso, Italy; tiziana.menegon@aulss2.veneto.it; 8 Hospital Management, Local Health Authority of Pescara, 65100 Pescara, Italy; fabrizio.cedrone@asl.pe.it; 9 Department of Biomedical Sciences for Health, University of Milan, 20133 Milan, Italy; vincenza.gianfredi@unimi.it

**Keywords:** social stigma, validation, Italy, surveys, questionnaires, mental health

## Abstract

Stigma negatively impacts individuals, families, and communities, affecting relationships, education, and employment and leading to an additional burden on mental health. Assessing public attitudes towards people with mental health conditions is crucial, especially in terms of public health. Therefore, the Attitudes to Mental Illness Questionnaire (AMIQ) was validated and adapted to the Italian cultural context. Translation followed four phases, involving bilingual speakers, comparison, back-translation, and expert review. In the pre-test phase, the questionnaire was administered to 21 participants anonymously. The validation test involved 213 subjects. Statistical analyses included exploratory and confirmatory factor analysis, and McDonald’s Omega and Cronbach’s alpha to assess the internal consistency. The results indicate good internal consistency (Omega = 0.71; Alpha = 0.72), and confirmatory factor analysis (CFI = 0.971) validated the questionnaire’s construct. The study’s findings align with the original validation, underscoring the questionnaire’s robustness. Overall, understanding public attitudes is crucial for public health interventions combating stigma and fostering positive attitudes.

## 1. Introduction

Attitudes towards people with mental conditions (PWMC) encompass a range of inclinations, from acceptance and tolerance to stigmatization. Stigma is defined as a deeply discriminating behavior that affects someone who has physical deformities, character flaws (such as mental health conditions), or beliefs or origins (e.g., nation, religion) [1]. The continuum between tolerance and stigma can significantly influence the willingness of PWMC to disclose their psychological problems and seek help [2,3,4,5]. Public health attitudes, in particular, exert a dual effect on PWMC. On one hand, these attitudes can directly influence how others, including the general population, healthcare professionals, family members, friends, and colleagues, interact with PWMC. This influence can manifest in providing support and care, offering new opportunities, or leading to avoidance and discrimination. Recognizing the potential impact of public health attitudes on PWMC is crucial, and effort should be made towards fostering a more inclusive and supportive society. For instance, PWMC could benefit from positive attitudes, such as supportive and inclusive behaviours. However, they may also encounter exploitation and exclusion from routine daily activities, such as job opportunities, due to discriminatory prejudices. On the other hand, PWMC may experience negative consequences as a result of self-limiting behavior. They might refrain from disclosing their symptoms out of fear of stigma, hindering their ability to express psychological distress and seek help [5,6].

A high prevalence of negative attitudes towards PWMC is reported not only among the general population [7,8,9], but also among healthcare professionals [10,11]. Although there is no apparent association between gender and attitudes, some evidence suggests that negative public attitudes are more prevalent among older individuals and those with lower levels of education [8]. This places an additional burden on overall mental health, leading to an increase in both direct and indirect costs [12]. As reported in 2022 by *The Lancet Commission on ending stigma and discrimination in mental health*, PWMC face a dual burden. Alongside the direct effects of their primary illness, they often endure severe consequences of stigma and discrimination, with stigma sometimes proving worse than the condition itself [13]. Stigma and discrimination negatively impact not only individual patients, but also families and communities, ranging from damaging marriage and family prospects to social exclusion in educational and work-related environments [14]. A persistent threat remains to the human rights of PWMC, including, but not limited to, loss of property, inheritance, or voting rights [15]. Furthermore, limited access to healthcare for non-mental health conditions is well-documented [16]. Stigma persists globally, across cultures and geographies. While evidence is solidifying in high-income countries, it is also emerging in low- and middle-income countries [13,15]. Moreover, despite increased mental health literacy, there has been insufficient improvement in social acceptance and a lack of reduction in stigma and discrimination towards PWMC, as indicated by multiple studies. For certain conditions, such as schizophrenia, public attitudes have even worsened, as noted by Schomerus and colleagues [2,17]. In recent years, several tools have been designed to assess stigma [18,19]. Among these, some have already been validated in an Italian population [20,21]. However, it is necessary to consider a broader range of aspects, including cultural and contextual aspects and attitudes towards PWMC, such as acceptance and tolerance. Furthermore, most of the questionnaires available for this purpose contain a high number of items which require a considerable amount of time to be addressed. In light of this, the *Attitudes to Mental Illness Questionnaire* (AMIQ) was formulated as a multidimensional tool used to quickly evaluate attitudes towards PWMC [22].

Assessing public attitudes towards people with mental health conditions is crucial for both understanding the prevailing sentiments among the population and evaluating the impact of public health interventions designed to contrast stigma and enhance positive attitudes compared to baseline values. However, considering the countries and cultural differences involved in the stigma of mental illness, context and cultural adaptation of available tools have become fundamental [23]. To our knowledge, no Italian validation studies regarding the AMIQ have been published thus far. As a result, our study aimed to translate, adapt, and validate the AMIQ for the Italian population.

## 2. Materials and Methods

### 2.1. Study Design

This is a cross-sectional study aimed at translating and validating the Italian version of the Attitudes to Mental Illness Questionnaire (AMIQ). The AMIQ is a self-assessment questionnaire consisting of 5 items that measure an individual’s attitude toward mental illness. It was developed by Cunningham, Sobell, and Chow [24] and validated by Luty and colleagues [22]. Participants are required first to read a vignette. The validation study makes available various vignettes describing individuals with different backgrounds: a person that injects heroin daily (John), a person with depression and a recent history of a suicide attempt (Tim), a person who has been drinking for 5 years (Steve), a convicted criminal (Robert), a person living with diabetes (Peter), a person with schizophrenia and a history of forced hospitalization (Michael), and a practicing Christian. For the purpose of our study, we chose to select the first vignette to gain a preliminary understanding of the feasibility and validity of administering the AMIQ in an Italian population. After reading the vignette, the participants can respond to each of the 5 items using a 5-point Likert scale, with scores ranging from a minimum of −2 to a maximum of +2. Items 1, 4, and 5 are reversed. “I don’t know” and “Neutral” responses are coded as 0. The scores for the 5 items are summed to provide a total score that ranges from −10 to +10. Higher scores indicate a more positive attitude. The AMIQ was translated and pre-tested on a small sample of 21 subjects to explore the latent structure of the questionnaire. Lastly, a validation test on a greater sample was performed to confirm the latent structure identified in the pre-test sample.

### 2.2. Translation

The translation procedure conformed to international guidelines for culturally adapting self-report measures [25]. In detail, four distinct phases were followed. The first phase involved direct translation of the questionnaire by two bilingual Italian native speakers, independently. The two translators had different backgrounds, one coming from the humanities and the other from the medical sciences. In the second phase, the two translations were compared, and through comparison and discussion with a third member of the research team, a third version was prepared. During the third phase, this consensus version was back-translated into English. The back-translation was carried out by two Italian-English translators with humanities backgrounds who were not informed about the concepts being explored. In the fourth phase, the two English versions (the original and back-translated versions) were then reviewed by an expert committee to identify any potential discrepancies and approve the final version of the translation.

### 2.3. Pre-Test

During the pre-test validation process, we administered the pre-final version of the questionnaire to a sample of 21 participants, selected using snowball sampling, among personal and non-personal contacts. Data were collected anonymously, between the 10th and 15th of May 2023 through an online survey developed using Google Forms (©2022 Google, Mountain View, Santa Clara, CA, USA) and administered online through social networks. Participants were instructed on the study’s aim and methods regarding how to fill out the questionnaire. The pre-final version of the questionnaire was followed by an open-ended question eliciting difficulties observed during the competition and suggestions to improve the clarity of the questionnaire.

### 2.4. Validation Test

Based on the results of the pre-test phase, the final version of the questionnaire was administered to a sample of 213 subjects recruited during the Networking Event titled “Mental Health for All”, organized by the “Public Mental Health” Working Group of the Italian Society of Hygiene, Preventive Medicine and Public Health during the European Public Health Week (22–26 May 2023) promoted by the European Public Health Association (EUPHA). The anonymous and voluntary survey, developed using Google Forms, was integrated into the event’s registration form. Informed consent was required from each participant.

### 2.5. Statistical Analysis

During the pre-test phase, Bartlett’s test of sphericity was performed to assess the suitability of the data for factor analysis. Then, exploratory factor analysis (EFA) using varimax rotation was carried out to extract two latent factors. A factor loading higher than or equal to 0.30 was used as a cut-off to assign the item to the factor itself. McDonald’s Omega was used to assess the internal consistency of the questionnaire in the pre-test sample; an omega higher than 0.7 was considered to represent good reliability.

During the validation test phase, Bartlett’s test of sphericity was performed before the confirmatory factor analysis (CFA) with robust weighted least square estimation using a model with the two factors extracted from the previous EFA. The following optimal cut-off goodness-of-fit indices were used: Comparative Fit Index (CFI) and Tucker–Lewis Index (TLI) > 0.9, root mean square error of approximation (RMSEA, and its relative 90% CI) < 0.08, and standardized root mean square residual (SRMR) < 0.08. Moreover, McDonald’s omega and Cronbach’s alpha were used to assess the internal consistency of the questionnaire in the validation sample; an alpha and omega higher than 0.7 were considered good reliability.

The 95% confidence intervals (95% CI) were calculated using a non-parametric bootstrap method with 10,000 replicates. A comparison between the stigma scores of the original validation study and our samples was conducted to confirm the robustness of our results. A *p*-value lower than 0.05 was considered significant.

## 3. Results

### 3.1. Pre-Test Results

During the pre-test phase, 21 volunteers agreed to fill in the questionnaire. Approximately half of the sample were women (n = 11, 52.4%), and the most common age group ranged from 35 to 54 years old (n = 10, 47.6%). In our sample, 90.4% agreed or strongly agreed that injecting heroin daily for one year would damage the subject’s career. Additionally, 57.1% disagreed or strongly disagreed with feeling comfortable working alongside the injecting subject or inviting him/her to a dinner party (52.4%). Meanwhile, the majority of the sample (61.9%) believed that it would be quite likely or very likely for the subject’s wife to leave him or to get in trouble with the law (90.5%). Detailed results are presented in Figure S1.

Bartlett’s test of sphericity was significant (*p* = 0.008), indicating the adequacy of factor analysis. Exploratory factor analysis showed that the items “Do you think that this would damage John’s career?”, “How likely do you think it would be for John’s wife to leave him?”, and “How likely do you think it would be for John to get in trouble with the law?” comprised one latent factor that was named “Prejudice”, while the other two comprised the second one, named “Discomfort”. Factor loadings are presented in Table 1.

McDonald’s omega was 0.82, indicating good internal consistency of the questionnaire.

### 3.2. Validation Test Results

During the validation phase, all 213 participants who registered for the event outlined in Section 2.4 filled in the questionnaire. Most of them were women (n = 139, 65.3%), and the most represented age category was those below 23 years old. Resident physicians and bachelor’s and master’s students were the main roles declared by participants (n = 82 (38.5%), n = 72 (33.8%), and n = 48 (22.5%), respectively), and most of them were involved in healthcare studies (n = 151, 70.9%). Socio-demographic characteristics are presented in Table 2.

**Figure 1:**
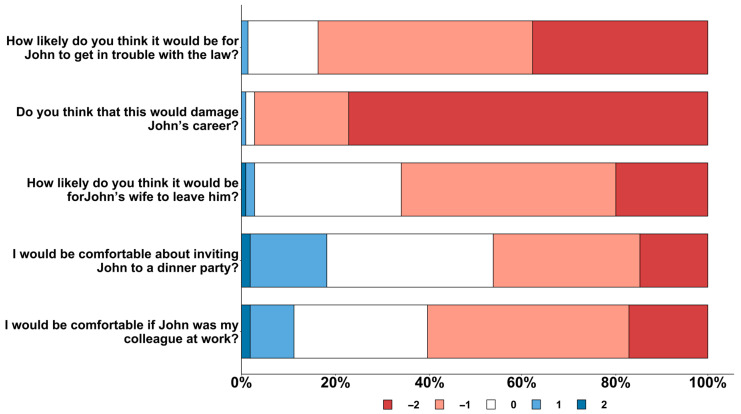
The distribution of the respondents’ answers per each item. For each question, −2 points correspond to the answer that reflects the highest level of stigma. Conversely, 2 points correspond to the answer that reflects the lower level of stigma.

In our sample for the validation test (n = 213), 97.3% agreed or strongly agreed that the subject’s professional prospects would be adversely affected by injecting heroin on a daily basis for one year. Furthermore, 60.1% disagreed or strongly disagreed regarding their comfort working alongside the injecting individual or inviting them to a dinner gathering (46.1%). At the same time, the majority of the respondents (67.7%) believed that it would be quite likely or highly likely for the subject’s spouse to leave them or for the subject to become entangled with the legal system (83.6%). Results are shown in Figure 1. Our sample’s stigma level was −4.79 (95% IC: −5.13; −4.46), indicating an intermediate-to-high level of stigma.

McDonald’s omega was 0.71, while Cronbach’s alpha was 0.72, indicating good internal consistency of the questionnaire.

### 3.3. Confirmatory Factor Analysis

**Figure 2:**
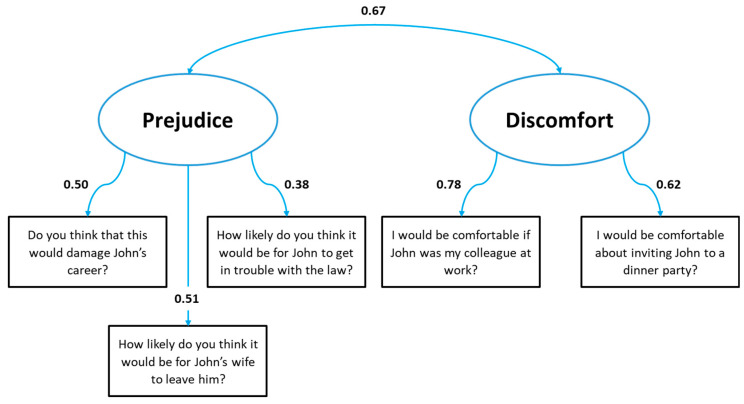
Model with standardized factor loadings.

Bartlett’s test of sphericity was significant (*p* < 0.001), indicating the adequacy of the data for factor analysis. The confirmatory factor analysis using the two latent factors model extracted from the EFA showed optimal goodness-of-fit indexes: the CFI was 0.971, the TLI was 0.927, the RMSEA was 0.059 (90% CI: 0.000; 0.117), and the SRMR was 0.042, indicating a good model to explain our constructed questionnaire. The model and the relative standardized factor loadings are shown in Figure 2.

### 3.4. Comparison of Stigma

**Figure 3:**
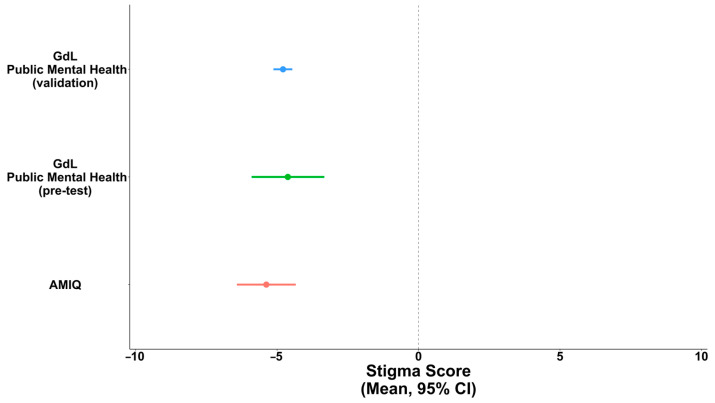
Comparison between the means and 95% confidence intervals between survey responses.

The stigma level in our validation sample [−4.79 (95% CI: −5.13; −4.46)] was comparable to that reported in the original validation of this questionnaire [−5.38 (95% CI: −5.90; −4.86)] [22]. The mean (point) and relative 95% CI (line) of each study are shown in Figure 3.

## 4. Discussion

In our study, we translated, adapted, and validated the AMIQ for the Italian population. The validation analysis demonstrated strong internal consistency. Using exploratory factor analysis with a small sample size, particularly when the number of participants is below 50, is a recognised technique for estimating latent factors [26]. This approach is time-saving and improves efficiency in the validation process of a questionnaire that has already been validated in another language. Subsequently, a confirmatory factor analysis could be conducted to assess the accuracy of the EFA results. In our study, the validity of a two-latent-factor model identified in the exploratory factor analysis was confirmed through confirmatory factor analysis. The goodness-of-fit indices met all the cut-off criteria outlined in the Methods section, affirming the validity of the two-latent-factor model identified in the exploratory factor analysis.

The main strength of the five-item AMIQ lies in its ability to combine conciseness and ease of administration with good psychometric properties. As a vignette-based questionnaire, it can overcome barriers associated with sensitive topics or the need for a high level of mental health literacy. Indeed, participants are not required to possess in-depth knowledge of the topics, making it accessible to individuals without experience, potentially at any age [27].

The original five-item AMIQ in English showed high content validity and reliability, as reported by Luty and colleagues [22]. Differently from the original validation study that used a one-factor model, in our validation, we adopted a two-latent-factor model. This divergence has roots in the different sample populations considered. Our analysis was conducted on a younger population, as 60% of our participants were below 28 years old, while in the validation study, the mean age was 46. Moreover, most of our participants were students of the health area, while in the original validation, 55% were paid employers and 37% were retired. Similarly to the English version’s validation study, our Italian version showed good internal consistency (McDonald’s omega = 0.71 and Cronbach’s alpha = 0.72). The robustness of our results was further confirmed by comparing the stigma scores in our study with those recorded in the validation study for the English version. In our study, stigma scores were substantially similar to those obtained in the English validation study for the same vignette: −4.79 (standard error, s.e. = 0.17) and −5.38 (s.e. = 0.53), respectively. In the AMIQ validation study, participants were presented with various vignettes featuring individuals with different backgrounds: a person with depression and a recent history of a suicide attempt (Tim), a person who had been drinking for 5 years (Steve), a convicted criminal (Robert), a person living with diabetes (Peter), a person with schizophrenia and a history of forced hospitalization (Michael), and a practicing Christian. The mean stigma score varied widely among scenarios. In particular, the least stigmatized (accepted) individuals were the practicing Christian (mean = 5.86) and Peter with diabetes (mean = 5.62), while the most stigmatized people were Robert, the criminal (mean = −5.90), and John, a person who injected heroin daily for 1 year (mean = 5.38). Furthermore, considering the person with depression and person with schizophrenia, the stigma mean scores were close to zero: 2.35 for Tim and −1.86 for Michael. Despite both conditions being treatable by a psychiatrist, the person who attempted suicide was more accepted than the one who was compulsorily hospitalized due to hallucinations caused by his mental disturbance. These different behavioral responses of the population toward those with mental illness is interesting: the ones who attempt suicide are accepted and helped, while the ones who have bizarre behaviors due to mental disorders tend to be ostracized and stigmatized.

Our validated Italian version of the AMIQ serves as an alternative to questionnaires already validated in Italian that assess stigma towards mental illness. Among these are the Italian version of the Attribution Questionnaire-27 (AQ-27-I) and the Opening Minds Stigma Scale for Healthcare Providers (OMS-HP) [20,21]. Similarly to the AMIQ, the AQ-27-I proposes a brief scenario followed by 27 questions that evaluate different dimensions of stigma, such as personal responsibility, pity, help, anger, coercion, and segregation. Although characterized by satisfactory internal consistency and stability, the low Cronbach’s alpha of some factors such as responsibility, anger, coercion, and avoidance and the amount of time needed to respond to a high number of items are some disadvantages to take into consideration when deciding which questionnaire to adopt to measure stigma attitudes. Like in our validation study, the Italian version of the OMS-HP was validated in a sample of Italian students of the health sector. Although this 12-item questionnaire requires more time to fill out when compared to the AMIQ, it shows very good validity and stability, and it was particularly designed to assess stigma in healthcare providers.

As outlined in many studies, healthcare professionals ought to serve as a powerful asset in combating the stigma associated with mental illness [28]. In light of this, our results are even interesting when considering the population among whom the studies were conducted. Actually, it should be noted that the participants were medical students and researchers [26], nurses [27], and medical assistants [29]. Our results are disheartening given the anticipated ability of participants to demonstrate sensitivity towards mental health matters. Similarly, most of our participants were enrolled in healthcare studies, and a majority of them were younger than 23 years old. These students represent the future healthcare workers (HCWs) within the next few years. A systematic review published in 2013, which included 28 studies, revealed that HCWs generally harbor negative attitudes towards patients with substance use disorders. Some contributing factors to this phenomenon include inadequate training and insufficient education for HCWs dealing with such patients [29]. Proposed solutions to address the stigmatization in the healthcare environment involve providing more targeted education and training before their entry into the workforce, incorporating curricular internships, and offering organizational support and counseling opportunities for HCWs working with these patients. Ultimately, such programs may enhance the quality of healthcare delivery for these patients [30,31,32]. In this respect, previous studies have administered the AMIQ before and after completion of an educational intervention, proving how the AMIQ can vary following an educational intervention [32,33]. Actually, in all these studies, the pre-intervention phase yielded poor results, similarly to our own study, indicating a negative attitude among the sample population.

This is significant as it indicates the tool’s usefulness in evaluating the efficacy of tailored programs and interventions targeted at healthcare professionals and the broader population. Concerning the low scores achieved on the AMIQ among healthcare worker populations, Chandramouleeswaran and colleagues undertook a study with postgraduate doctor trainees [34]. The study suggested that adequate training in psychiatry during university has a positive impact on attitudes towards individuals with mental health conditions compared to family or previous experience of mental illness. Such training gives individuals the perception of possessing greater skills to manage patients or specific cases. This indicates that the capability to mitigate stigmatization among healthcare professionals who are not psychiatry specialists lies not in the qualification, but in the quality and comprehensiveness of university education regarding mental health.

### 4.1. Implication for Public Health Policies

Translating and validating a tool designed to assess attitudes toward mental health stigma, while also considering the cultural context, has important implications in terms of public health. Indeed, having a valid tool is extremely important for conducting assessments of the level of stigma towards mental health in the referenced population. This aspect is pivotal for policymakers who want to gain accurate insights into prevailing sentiments within a community. This cultural adaptation ensures that the assessment tool is sensitive to the nuances of local beliefs, values, and perceptions related to mental health. Deeply understanding the phenomenon is essential to designing, planning, and implementing public health interventions aimed to combat stigma, promote mental health awareness, and improve attitudes toward individuals with mental health conditions. Indeed, contrasting stigma is of paramount importance at each level. Limitations due to stigmatization also extend to social and cultural rights, imposing restrictions on education and employment opportunities. Furthermore, stigmatization often results in unhygienic living conditions and unhealthy physical and sexual practices. These inappropriate behaviors contribute to the spread of both communicable and noncommunicable diseases, resulting in worse health outcomes. Additionally, these behaviors have repercussions on civil and political rights, restricting the public lives of PWMC. The marginalization of PWMC significantly impedes the attainment of international development goals set by the World Health Organization (WHO) [15].

Comprehensive strategies for promoting mental health and preventing mental disorders should particularly emphasize antidiscrimination laws and information campaigns aimed at rectifying the stigmatization and human rights violations frequently associated with mental disorders [35]. In light of this, having validated tools for assessing stigma allows for evaluating its trends over time and verifying the potential effectiveness of interventions aimed at reducing the level of stigma in society, with the final aim of fostering a supportive environment for individuals dealing with mental health conditions. Additionally, using validated tools has several advantages in terms of accuracy, consistency, and credibility of the collected data, contributing to increasing effectiveness, as validated tools are proven to measure what they intend to assess. These are important aspects that should be considered when evidence-based decisions are needed.

### 4.2. Limitations and Strengths

Before generalizing, some limitations should be considered. First, a social desirability bias cannot be ruled out. It is possible that some respondents were more likely to express the general public’s opinion than their own, particularly on issues about which they may be ambivalent. However, previous research has shown that people respond to vignettes in much the same way as they would in a real-life situation and that respondents are less likely to give socially acceptable answers than when asked directly [36]. Secondly, the cultural background of part of the sample and the over-representation of younger age groups compared to the general population may limit the generalizability of the results. Moreover, despite the AMIQ being a vignette-based questionnaire that does not demand a high level of health literacy for comprehension, it is important to note that our validation sample mainly consisted of healthcare workers and health sciences students, which might constrain the extent to which we can generalize the validation results to the broader Italian population. Lastly, the validation population consisted predominantly of women (65.3%), a demographic composition that may have introduced a potential influence on our findings. It is noteworthy that certain studies exploring stigma within populations with similar mean ages have identified male gender as a factor that is positively correlated with higher levels of stigma [37,38]. While a full comprehension of the underlying reasons for this phenomenon remains elusive, a partial elucidation can be drawn from entrenched masculine norms—socially defined rules and expected behaviors linked to men and manhood within a specific culture [39]. Adherence to these rigid norms among males may contribute to the stigmatization of individuals with mental illnesses. The subtle imbalance in the representation of men and women in our validation sample, even if only marginally uneven, could potentially have influenced the validation construct or resulted in a distinct mean stigma score.

Despite these limitations, our study has notable strengths. It is the first to validate an Italian version of the AMIQ. This accomplishment creates opportunities for future researchers, enabling them to utilize this user-friendly questionnaire with just five questions. This tool provides an avenue to explore a topic that is gaining prominence within the scientific community. Moreover, the study adhered to a four-phase translation process characterized by a stringent methodology. Subsequently, the validation process encompassed a comprehensive analysis, employing statistical methodologies well-established in the scholarly literature for the validation of questionnaires. Specifically, the internal consistency analysis revealed high reliability, as indicated by McDonald’s omega value (omega = 0.71). Additionally, the confirmatory factor analysis showcased a favorable fit, confirming the underlying structure identified in the exploratory factor analysis. These results lend credence to the tool’s stability and consistency in measuring the intended constructs. Lastly, the methodological approach we used ensured a robust and rigorous validation framework in line with established standards in the field.

### 4.3. Future Perspectives

With the current work, we pave the way for more accurate and meaningful assessments of stigma surrounding mental health issues in Italy. The implications are far-reaching, extending to both research and practical applications. In research, this culturally validated tool opens avenues for more nuanced investigations into the nature and extent of mental health stigma within the Italian population. Researchers can delve deeper into the cultural factors influencing perceptions of mental health, contributing to a richer understanding of the complexities surrounding stigma. However, the validated tool bolsters the credibility of research endeavors and has practical implications for healthcare practitioners. With a validated tool at their disposal, professionals can now embark on more precise investigations into the prevalence and nature of mental health stigma in Italy. Furthermore, the tool’s validation enhances its potential for integration into routine mental health assessments, ensuring that stigma is consistently monitored and addressed within healthcare practices. In the broader context, the validation of the stigma assessment tool contributes to the global dialogue on mental health. Comparative studies and collaborations with countries that have validated similar tools could offer insights into shared challenges and best practices. Lastly, the AMIQ translation and validation hold the key to transformative changes in how society, healthcare systems, and policymakers approach and combat mental health stigma in the Italian context.

## 5. Conclusions

The present study provides robust evidence supporting the validity of the Italian version of the AMIQ as a reliable instrument for assessing attitudes towards mental health stigma. Our findings, derived from a rigorous validation process, offer substantial support for the tool’s robustness, confirming its applicability to the research, clinical, and policymaking sectors. To conclude, our study not only contributes to proving the validity of the Italian version of the AMIQ, but it provides valuable insights into the broader implications for public health policies and the importance of culturally sensitive and validated assessment tools.

## Figures and Tables

**Table 1:** Exploratory factor analysis loadings.

Question	Factor 1 (Prejudice)	Factor 2 (Discomfort)
Do you think that this would damage John’s career?	**0.517**	0.272
I would be comfortable if John was my colleague at work	0.303	**0.477**
I would be comfortable about inviting John to a dinner party	0.068	**0.995**
How likely do you think it would be for John’s wife to leave him?	**0.853**	0.037
How likely do you think it would be for John to get in trouble with the law?	**0.339**	0.297

Bold = statistical significance.

**Table 2:** Socio-demographic characteristics of the respondents.

Questionnaire (Total Respondents = 213)	n = 213
Sex	
Woman	139 (65.3%)
Man	67 (31.5%)
Prefer not to specify	7 (3.3%)
Age group	
<23	63 (29.6%)
23–25	39 (18.3%)
26–28	27 (12.7%)
29–31	22 (10.3%)
32–34	14 (6.6%)
35–54	44 (20.7%)
55–75	3 (1.4%)
>75	1 (0.5%)
Role	
Medical doctor in postgraduate training	82 (38.5%)
Healthcare worker	6 (2.8%)
PhD candidate and researcher	3 (1.4%)
Master’s or bachelor’s students	122 (57.3%)

## Data Availability

The authors can be contacted for information about the data presented.

## Supplementary Materials

The following supporting information can be downloaded at: https://www.mdpi.com/article/10.3390/healthcare12030395/s1, Figure S1: Distribution of the answers for each item of the questionnaire during the pre-test phase.
