# Supplementary material for: An Italian Validation of the 5-Item Attitudes to Mental Illness Questionnaire (AMIQ): A Useful Tool for Rapid Assessment of Stigma, Acceptance, and Tolerance
Source: Healthcare (Basel). 2024 Feb 3;12(3):395. doi: 10.3390/healthcare12030395 (PMC10855529; doi:10.3390/healthcare12030395)

Figure S1. Distribution of the answers for each item of the questionnaire during the pre-test phase

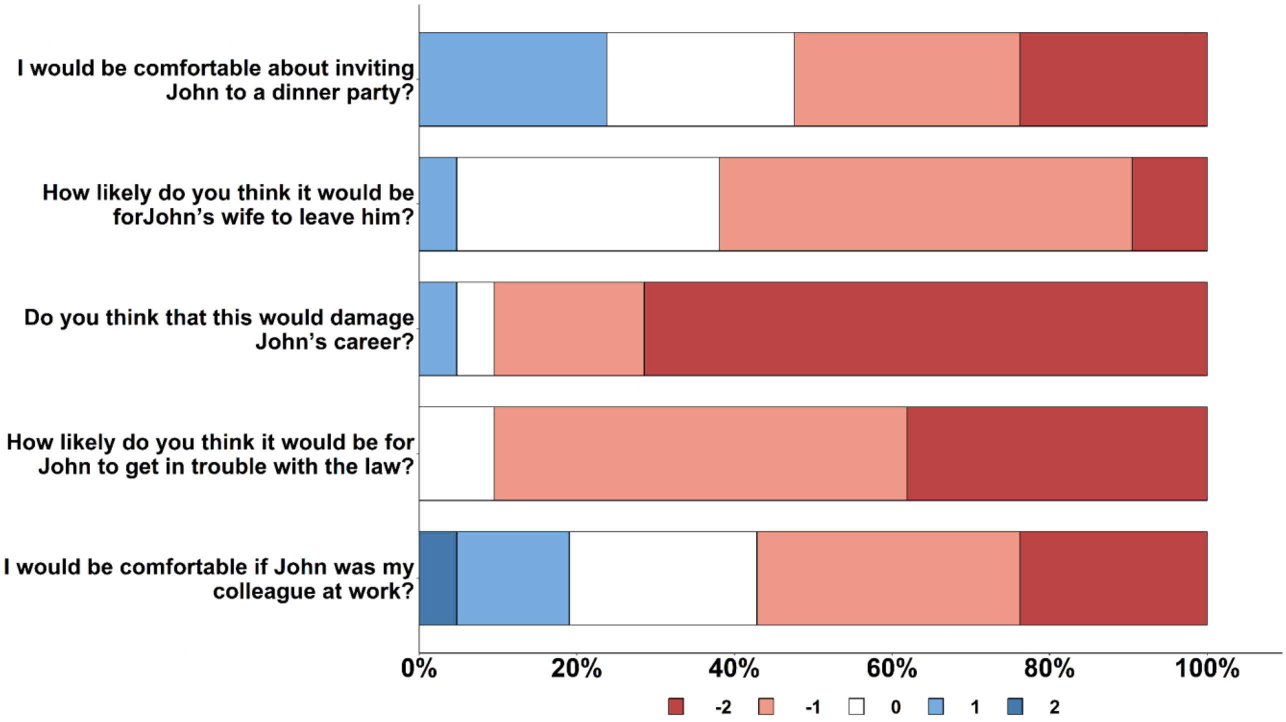

Supplement: Supplementary file 1 [file healthcare-12-00395-s001.zip › healthcare-2816466-supplementary.pdf]
